# Cardioprotective Effects of a Selective c-Jun N-terminal Kinase Inhibitor in a Rat Model of Myocardial Infarction

**DOI:** 10.3390/biomedicines11030714

**Published:** 2023-02-27

**Authors:** Mark B. Plotnikov, Galina A. Chernysheva, Vera I. Smol’yakova, Oleg I. Aliev, Tatyana I. Fomina, Lyubov A. Sandrikina, Irina V. Sukhodolo, Vera V. Ivanova, Anton N. Osipenko, Nina D. Anfinogenova, Andrei I. Khlebnikov, Dmitriy N. Atochin, Igor A. Schepetkin, Mark T. Quinn

**Affiliations:** 1 Department of Pharmacology, Goldberg Research Institute of Pharmacology and Regenerative Medicine, Tomsk National Research Medical Center, Russian Academy of Sciences, 634028 Tomsk, Russia; 2 Faculty of Radiophysics, National Research Tomsk State University, 634050 Tomsk, Russia; 3 Department of Morphology and General Pathology, Siberian State Medical University, 634050 Tomsk, Russia; 4 Department of Pharmacology, Siberian State Medical University, 634050 Tomsk, Russia; 5 Cardiology Research Institute, Tomsk National Research Medical Center, Russian Academy of Sciences, 634012 Tomsk, Russia; 6 Kizhner Research Center, Tomsk Polytechnic University, 634050 Tomsk, Russia; 7 Cardiovascular Research Center, Cardiology Division, Massachusetts General Hospital, Harvard Medical School, Charlestown, MA 02115, USA; 8 Department of Microbiology and Cell Biology, Montana State University, Bozeman, MT 59717, USA

**Keywords:** cardioprotective activity, c-Jun N-terminal kinase inhibitor, 11*H*-indeno[1,2-*b*]quinoxalin-11-one oxime, heart failure, infarct size, myocardial infarction, ischemia/reperfusion

## Abstract

Activation of c-Jun N-terminal kinases (JNKs) is involved in myocardial injury, left ventricular remodeling (LV), and heart failure (HF) after myocardial infarction (MI). The aim of this research was to evaluate the effects of a selective JNK inhibitor, 11*H*-indeno [1,2-*b*]quinoxalin-11-one oxime (IQ-1), on myocardial injury and acute myocardial ischemia/reperfusion (I/R) in adult male Wistar rats. Intraperitoneal administration of IQ-1 (25 mg/kg daily for 5 days) resulted in a significant decrease in myocardial infarct size on day 5 after MI. On day 60 after MI, a significant (2.6-fold) decrease in LV scar size, a 2.2-fold decrease in the size of the LV cavity, a 2.9-fold decrease in the area of mature connective tissue, and a 1.7-fold decrease in connective tissue in the interventricular septum were observed compared with the control group. The improved contractile function of the heart resulted in a significant (33%) increase in stroke size, a 40% increase in cardiac output, a 12% increase in LV systolic pressure, a 28% increase in the LV maximum rate of pressure rise, a 45% increase in the LV maximum rate of pressure drop, a 29% increase in the contractility index, a 14% increase in aortic pressure, a 2.7-fold decrease in LV end-diastolic pressure, and a 4.2-fold decrease in LV minimum pressure. We conclude that IQ-1 has cardioprotective activity and reduces the severity of HF after MI.

## 1. Introduction

Myocardial infarction (MI) is classified as the leading cause of global mortality from various types of cardiovascular diseases [1]. Heart failure (HF) is a severe consequence of MI associated with high mortality [2,3,4,5] and is characterized by a decrease in cardiac output due to left ventricular (LV) remodeling associated with an increase in infarct size, progressive LV dilatation, hypertrophy of non-infarct regions, cardiomyocyte loss, and fibrosis [6,7,8,9,10,11,12].

Recent studies have shown that the c-Jun N-terminal kinase (JNK) pathway is involved in myocardial ischemia/reperfusion (I/R) injury [13,14,15,16]. JNK plays an important role in the regulation of inflammation, apoptosis, necrosis, and several transcriptional and non-transcriptional processes involved in cardiomyocyte injury during I/R [15,17]. Increased activity of JNK signaling is evident during HF development [18,19,20,21]. Contribution of the JNK pathway to postinfarction HF pathogenesis has been convincingly demonstrated in experimental animals and in cell culture [14,15,16,21,22,23]. On the other hand, many questions regarding the causal relationships between JNK activation and cardiac dysfunction in HF remain unsolved [15]. Indeed, JNK activation after MI is bidirectional and involved both in MI pathogenesis and in compensatory, protective reactions after I/R occurs in the myocardium [15,24]. Furthermore, inhibition of different JNK isoforms has different effects on apoptosis in cardiomyocytes [25].

JNK suppression by peptide and synthetic inhibitors such as d-JNKI-1, AS601245, SP600125, SU3327, and SR 3306 early after I/R myocardial injury has been shown to decrease myocardial infarct size and attenuate cardiomyocyte apoptosis [26,27,28,29,30,31,32]. Modulation of certain signaling pathways associated with the JNK signaling system can limit HF development after MI, which eventually results in JNK inactivation early after I/R [33,34,35,36]. However, there is a lack of research focused on studying the effects of JNK inhibitors on HF development late after MI.

Although several JNK inhibitors with acceptable pharmacokinetics are currently available [37], the complete non-specific inhibition of all JNK isoforms would be inappropriate in the treatment of these diseases. Conversely, the selective downregulation of individual JNK isoforms (e.g., JNK3 expressed in the heart) or the targeting of specific molecular domains of JNK-dependent cascades involved in pathological signal transduction may be promising [38]. A search for highly selective and nontoxic inhibitors of the JNK pathway continues to identify agents with high therapeutic potential. Previously, we identified a selective JNK inhibitor, 11*H*-indeno[1,2-*b*]quinoxalin-11-one oxime (IQ-1), with potent anti-inflammatory activity [39]. The aim of the present work was to study the effects of IQ-1 on HF development with the analysis of systemic hemodynamic parameters, cardiac hemodynamics, and postinfarction fibrosis in rats 60 days after I/R.

## 2. Materials and Methods

### 2.1. Animals

This study was performed in accordance with the EU Directive 2010/63/EU concerning the protection of animals used for scientific purposes and approved by the Animal Care and Use Committee of the Goldberg Research Institute of Pharmacology and Regenerative Medicine, Tomsk NRMC (Protocol No. 187092021 from 11.10.21). Experiments were performed on 79 adult male Wistar rats (280–300 g) obtained from the Department of Experimental Biological Models of E.D. Goldberg Institute of Pharmacology and Regenerative Medicine. Rats were housed in groups of five animals per cage (57 × 36 × 20 cm) under standard vivarium conditions (ambient temperature of 22 ± 2 °C, relative humidity of 60%, light/dark period of 12/12 h/day) in cages with sawdust bedding and provided with standard rodent feed (PK-120-1, Ltd., Laboratorsnab, Moscow, Russia) and ad libitum water access.

### 2.2. Equipment

Equipment included a rodent ventilator model 7125 (UGO Basile, Gemonio, Italy), a temperature control unit HB 101/2 (Spain), a homeothermic blanket control unit (Harvard Apparatus, Holliston, MA, USA), a computer electrocardiograph Poly-Spector-8/L (Neurosoft, Ivanovo, Russia), an MP150 high-speed data acquisition system with ECG100C and EBI100C amplifiers (Biopac Systems, Inc., Goleta, CA, USA), a pressure-measuring unit with a TSD282 micro pressure sensor (OpSens, Quebec, Quebec, Canada), a Tissue Matrix RBM2000C (ASI Instrument, Eugene, OR, USA), a CO_2_ euthanasia device (Open Science, Krasnogorsk, Russia), a light microscope (Axio Lab. A.1, Carl Zeiss, Jena, Germany) with Axio Cam ERC 5S and electronic scales BL-22OH (Shimadzu, Kyoto, Japan), and a freezing microtome (MZ-2, Kharkov Plant of Medical Equipment, Kharkov, Ukraine).

### 2.3. Chemicals and Drugs

The following chemicals and drugs were used: propofol-Lipuro (B. Braun Melsungen AG, Melsungen, Germany), diethyl ether for anesthesia (Kuzbassorghim, Kemerovo, Russia), Tween 80 (Merck, Darmstadt, Germany), 2,3,5-triphenyl tetrazolium chloride (TTC) (Sigma, St. Louis, MO, USA), thiopental sodium (Syntez, Kurgan, Russia), 10% neutral formalin, embedding medium Histomix, Vitrogel, Masson Trichrome Stain Kit, picrofuchsin solution for Van Gieson staining (BioVitrum, St. Petersburg, Russia), ethanol (Konstanta-Farm M, Moscow, Russia), and o-xylene (EKOS-1, Moscow, Russia).

### 2.4. Study Compound

IQ-1 was synthesized, as described previously [40]. The chemical structure was confirmed by mass spectrometry and nuclear magnetic resonance. Purity of the sample was 99.9%.

### 2.5. Experimental Protocol

**Figure 1:**
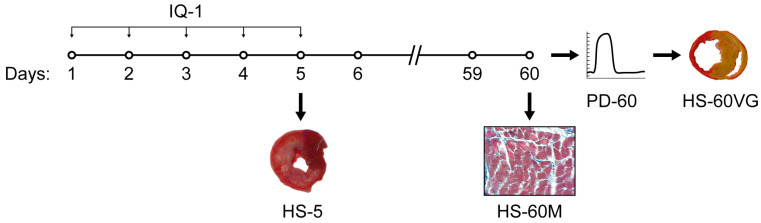
Schematic representative of experimental protocol. HS-5, illustration of a heart section on day 5 after MI (TTC staining); PD-60, illustration of pressure dynamics in LV on day 60 after MI; HS-60VG, illustration of heart section on day 60 after MI (Van Gieson’s staining); HS-60M—illustration of heart section on day 60 after MI (Masson’s staining).

The schematic protocol of the study is presented in Figure 1. Animals were randomly assigned to 3 groups: sham-operated (I; n = 17), control group (II; n = 33/24: total number/survived animals), and IQ-1-treated experimental group (III; n = 29/24). Rats in the control and experimental groups received temporary ligation of the left coronary artery; rats in the sham-operated group underwent surgery without coronary artery ligation. Rats in the experimental group received 25 mg/kg IQ-1 intraperitoneally (*i.p*.) as a suspension in 2 mL of sterile 0.9% NaCl with 20 µL of Tween 80 during ischemia (30 min prior to reperfusion), and then once daily for 4 subsequent days. Rats in groups I and II received *i.p.* 2 mL of sterile 0.9% NaCl with 20 µL of Tween 80 following the same scheme.

Five animals from group I, eight animals from group II, and eight animals from group III were randomly selected on day 5, three hours after the last IQ-1 injection. The animals were euthanized in a CO_2_ chamber, the hearts were removed, and morphological studies were performed. Sixty days after I/R, five animals were randomly selected from each group (groups I, II, and III) and euthanized in a CO_2_ chamber, and the hearts were removed and fixed in 10% formaldehyde solution for 24 h for further morphological studies. The rest of the animals from groups I, II, and III were subjected to general anesthesia by brief diethyl ether inhalation, then catheter implantation in the right femoral vein, and anesthesia with sodium thiopental at a dose of 35 mg/kg/h. Thoracic bioimpedance magnitude was registered to assess stroke volume, and an electrocardiogram was recorded. Next, a TSD282 micro pressure sensor was injected into the right common carotid artery to measure the mean arterial pressure and heart frequency. This sensor was held in the cavity of the left heart for the measurement and calculation of contractile function indices. At the end of the experiments, after the registration of the parameters of systemic and cardiac hemodynamics, the animals were euthanized with inhaled CO_2_, and the hearts were removed for morphological examination.

### 2.6. Model of Myocardium I/R

I/R of the myocardium was achieved, as described previously [41]. Briefly, after short-term anesthesia with diethyl ether, the animals were implanted with a catheter in the left femoral vein to provide anesthesia with propofol. Propofol was administered intravenously at a dose of 10 mg/kg/h. During the surgery, the rectal temperature of the animals was maintained in the range of 37 ± 0.5 °C using a homeothermic blanket control unit. After reaching the surgical stage of anesthesia, the animals were intubated without compromising the integrity of the trachea and connected to a ventilator. After thoracotomy and pericardiotomy, the left coronary artery was ligated with an atraumatic needle and 4-0 silk suture at the level of the *auricula sinistra* lower edge without disrupting the cardiac topography in the chest, in accordance with the Kogan method [42]. To achieve controlled occlusion, the ends of the ligature were passed through a 10 mm long, 2 mm diameter polyethylene tube. The wound was closed with clamps to restore the chest wall integrity, and the end of the polyethylene tube with the ligature sutures was removed. Occlusion of the coronary artery was performed by stretching the ends of the ligature and fixing them with a clamp. To verify coronary artery occlusion, the electrocardiogram was monitored with three standard leads using a Poly-Spector-8/L computer electrocardiograph. To perform reperfusion, the ends of the ligature were released from tension by removing the clamp. The duration of the left coronary artery occlusion was 45 min. The polyethylene tube and ligature were removed from the chest cavity; the wound surface was rinsed with sterile saline, and the muscles were sutured layer-by-layer to seal the chest. The catheter was removed from the vein. The sham control animals were subjected to the entire surgical procedure, and a silk suture was passed beneath the coronary artery, but the coronary artery was not ligated. After spontaneous breathing was restored, the animals were disconnected from the ventilator and extubated. The animals were returned to their cages with free access to water and food after recovery from anesthesia.

### 2.7. Assessment of Cardiac Parameters

Experiments were performed 60 days after MI to assess systemic and cardiac hemodynamic parameters. To achieve this, the animals were reanesthetized with diethyl ether, and a catheter was implanted into the femoral vein for anesthesia with sodium thiopental (dose of 35 mg/kg/h). During the study, the body temperature of the animals was maintained at 37 ± 0.5 °C using a homeothermic blanket control unit. After reaching the surgical stage of anesthesia, the heart rate (min^–1^) was determined by an electrocardiogram, and stroke volume (mL) was assessed by impedancemetry using an MP150 Biopac electrophysiological apparatus. Cardiac output (mL × min^–1^) was calculated using the formula:

Cardiac output = stroke volume × heart rate.

The left ventricle was accessed through the right common carotid artery. The parameters of cardiac contractile function were assessed based on the dynamics of intracardiac pressure using a TSD282 sensor and an intracardiac pressure-measuring device. Based on the pressure curve in the LV cavity, the following parameters were determined: LV systolic pressure, LV end-diastolic pressure, minimal pressure in the LV (LVP_min_), contractility index (s^–1^), and the maximum rates of the rise and the fall of ventricular pressure (+dP/dt_max_ and −dP/d_tmax_).

### 2.8. Assessment of Infarct Size

The MI area was determined in several rats from groups I, II, and III at 5 days after I/R. The hearts were removed from the animals after euthanasia. The atria and great vessels were removed. The heart was frozen at –12 °C for 2 h so that it could be cut more easily. The heart was cut into slices (1 mm thick) using an RBM2000C tissue matrix, and the sections were placed on glass slides. The slide sections were stained with a 1% solution of TTC. We used a 2-part buffer system consisting of 77.4% NaH_2_PO_4_ (0.1 mol/L) and 22.6% Na_2_HPO_4_ (0.1 mol/L), pH 7.4. The slices were incubated in the solution at 37 °C for 10 min to detect infarct zones based on the level of dehydrogenase activity; segments with red staining were designated as viable, and those without staining were designated as non-viable (infarcted). The stained sections were covered with glass slides, and the stained side of the sections was scanned on an HP Scanjet 3770 at 600 dpi, and the images were saved in .tiff format. The acquired images were processed using Adobe Photoshop 6.0. The areas of myocardial infarct zones (non-stained regions on sections) and the entire LV myocardium were calculated. The myocardial infarct area is expressed as a percentage (%) of the entire LV myocardial area.

### 2.9. Morphometric Assessment of Relative Sizes of Scar and LV Cavity and the Entire LV Myocardial Area

Sixty days after I/R, rat hearts were cut into a series of 500 μm thick cross-sections using a freezing microtome. Pairs of neighboring cross-sections were placed onto the corresponding pairs of glass slides. Glass slide 1 from each adjacent pair was stained with 1% TTC solution, as described above, for morphometric assessments of scar size, LV cavity area, and total LV myocardium area. Glass slide 2 from the pair was used to assess the area of mature connective tissue as follows: the sections of the heart were fixed on the slides, dehydrated with 95% ethanol, and stained with Van Gieson picrofuchsin for 2–5 min at 25 °C. The stained sections were covered with coverslips and scanned at 600 dpi. The images were saved in .tiff format and processed using Adobe Photoshop 6.0. Scar size was calculated as a percentage of the total LV myocardium area on TTC-stained sections. The total LV myocardium area and mature connective tissue area were measured on cross-sections stained with Van Gieson picrofuchsin. The area of mature connective tissue was expressed as a percentage of the total LV myocardium area.

### 2.10. Morphometric Assessment of the Development of Interstitial Fibrosis in the Distant Myocardium (Interventricular Septum)

The middle third of each LV was isolated and embedded in paraffin after formalin fixation to assess the development of interstitial fibrosis in areas remote from the MI focus. Thin, 5 μm LV transverse sections were prepared with Masson’s trichrome staining. The Masson-stained LV sections were photographed in 10 random visual fields in the area of the interventricular septum under an AxioLab.A1 microscope with an AxioCam ERc5s camera. A researcher unaware of the group assignment of the microphotographs assessed the area of intermuscular connective tissue in pixels using GIMP 2 software, and the area of connective tissue was calculated (in %) considering the number of pixels in the standard field of view.

### 2.11. Statistical Analysis

Statistical analysis was performed with Statistica 8.0 software. All results are expressed as the mean ± SEM. Group variation was assessed with the Kruskal–Wallis test. Significant differences were assessed by Fisher’s exact test for mortality and by the Mann–Whitney U test for all other parameters. The compliance of the sample with a normal distribution was evaluated with Shapiro–Wilk’s W test. Values were considered statistically significant when *p* was <0.05.

## 3. Results

### 3.1. Effect of IQ-1 on Survival Rates in Rats 60 Days after MI

All animals in the group of sham-operated rats survived for 60 days. In the control group, 7 out of 33 animals died within the first 24 h after MI; 2 animals died later, including one animal after 3 days and another 1 after 10 days. The survival rate 60 days after MI was 73% in the control group. In the experimental group, 4 out of 29 animals died in the first 24 h after MI, and 1 more animal died 30 days after reperfusion; the survival rate was 83%. The survival rate among animals in the IQ-1-treated group was not significantly different from the corresponding value in the control group.

### 3.2. Effect of IQ-1 on the Size of Infarct Zones in the Myocardium of Rats during the Acute Period after I/R

**Figure 2:**
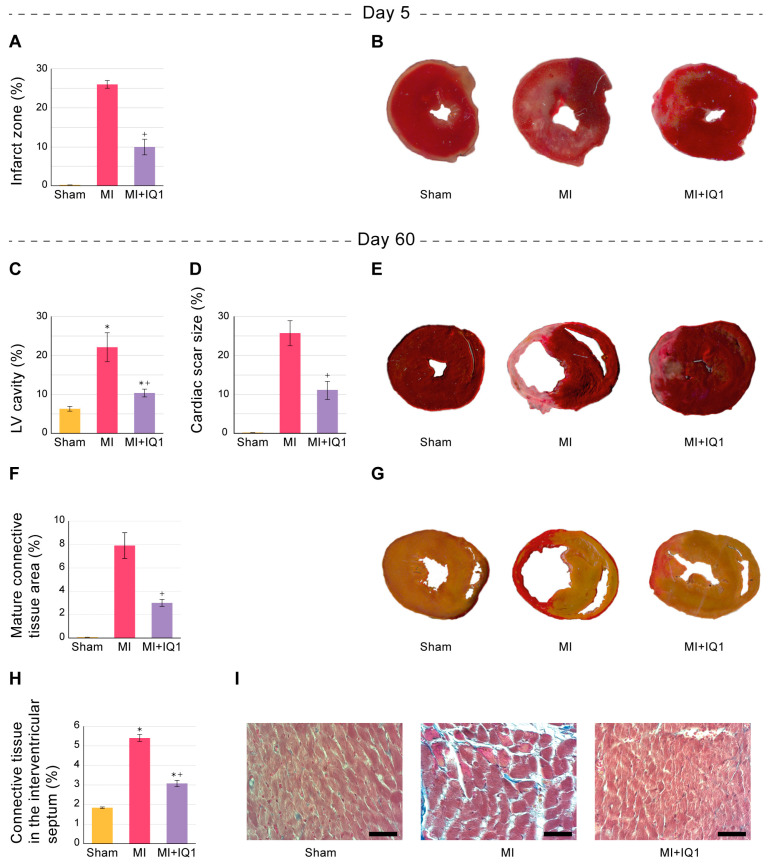
Effects of IQ-1 on myocardial infarct size. Panel (**A**): % of the entire LV myocardial area with representative heart section images (Panel (**B**)) from rats on day 5 after MI. Panel (**C**): LV cavity size (% of the entire LV myocardial area) and the area of scar zone (Panel (**D**), % of the entire LV myocardial area) with representative heart section images (Panel (**E**)). Panel (**F**): area of mature connective tissue (% of the entire LV myocardial area; Van Gieson’s staining) with representative heart section images (Panel (**G**)). Panel (**H**): connective tissue area in the interventricular septum (% of the entire LV myocardial area; Masson’s staining) with representative histological images (Panel (**I**), scale = 50 µm) in rats on day 60 after MI. * *p* < 0.05 as compared with sham-operated animals; ** *p* < 0.05 as compared with control animals.

The size of the MI zone was 26 ± 1% of the entire LV myocardial area in the control group of animals. The size of the MI zone in the group of animals treated with IQ-1 was 10 ± 2% of the entire LV myocardial area, which was 62% lower than the corresponding value in the rats in the control group (Figure 2A,B).

### 3.3. Effect of IQ-1 on Systemic Hemodynamic Parameters in Rats 60 Days after MI

A 32% decrease in stroke volume was observed in control group rats 60 days after MI compared with sham-operated animals, which, in the absence of changes in the heart rate, resulted in a 33% decrease in cardiac output (Table 1). A statistically significant 33% increase in stroke volume and 40% increase in cardiac output was observed in rats in the IQ-1-treated group 60 days after MI compared to the corresponding values in control rats, without significant changes in the heart rate (Table 1).

### 3.4. Effect of IQ-1 on Cardiac Contractility Parameters 60 Days after MI

The control group showed impaired myocardial contraction–relaxation compared with the corresponding values in sham-operated rats 60 days after MI (Table 1). +dP/dt_max_ and the contractility index decreased by 36% and 34%, respectively. Aortic pressure in control rats was 85% of that in the sham-operated group. All rats in the control group developed pronounced diastolic dysfunction in comparison with sham-operated animals: –dP/dt_max_ decreased by an average of 48%, LV end-diastolic pressure in control animals increased by 9.4-fold, and LVP_min_ increased by 11.8 mm Hg.

Rats in the IQ-1-treated group showed a significant improvement in myocardial contractile function, resulting in a 35% increase in +dP/dt_max_ and a 34% increase in the contractility index 60 days after MI compared to the corresponding values in the rats of the control group. Aortic pressure increased by 12% relative to that in the control group. There was also an improvement in myocardial relaxation, with a 48% increase in –dP/dt_max_. LV end-diastolic pressure and LVP_min_ decreased by 3.4- and 12.5-fold, respectively (Table 1).

### 3.5. Effect of IQ-1 on Morphological Changes in Rat Myocardium 60 Days after MI

The LV cavity was on average 6 ± 1% of the entire surface area in the group of sham-operated animals 60 days after surgery. During this period, the control animals demonstrated a significant expansion of the LV cavity by an average of 3.5-fold in comparison with that in rats in the sham-operated group (Figure 2C,E). An aneurysm of the anterior LV wall developed at the site of massive transmural MI in 3 out of 11 control rats, and the LV cavity in these rats increased by 4.8–7.6-fold compared with the corresponding value in sham-operated rats. The scar size area in control rats was on average 26 ± 3% of the entire LV myocardial area (Figure 2D,E).

Van Gieson-stained sections of the heart showed the formation of mature connective tissue area predominantly in the central sections of the scar. The area of mature connective tissue was 7.8 ± 1.1% of the entire LV myocardial area (Figure 2F,G). In contrast, there was a significant decrease in postinfarction LV remodeling in animals treated with IQ-1. The LV cavity size was 10 ± 1% of the entire surface area, which was 2.1-fold lower than the LV cavity size in control animals (Figure 2C,E). There were no animals with an aneurysm of the anterior wall or a significant expansion of the LV cavity. A significant decrease in scar size to 11 ± 2% of the entire LV myocardial area was observed in rats in the IQ-1-treated group (2.3-fold less than in the control group) (Figure 2D,E). The area of mature connective tissue was 3.0 ± 0.3% on average of the entire LV myocardial area, which was 2.6-fold less than that in the control group (Figure 2F,G).

Evaluation of interstitial fibrosis development in the interventricular septum in sham-operated rats showed that the myocardium in this zone contained 1.8 ± 0.1% connective tissue. The content of connective tissue significantly increased by 2.9-fold to 5.4 ± 0.2% in rats in the control group. In IQ-1-treated rats, connective tissue was decreased to 3.1 ± 0.2%, which was 43% lower than that in the control group (Figure 2H,I). No macrophage infiltration was found in the histological images. This is likely because the acute inflammatory response occurs in the myocardium during the first days after MI and would be resolved by the time of our analysis at 60 days [43].

## 4. Discussion

JNKs belong to the family of mitogen-activated protein kinases that are activated in response to a variety of stress factors, such as ultraviolet radiation, oxidative stress, heat and osmotic shock, the administration of protein synthesis inhibitors, and I/R [17,27,44,45,46]. The JNK family includes 10 isoforms encoded by three genes: JNK1 (4 isoforms), JNK2 (4 isoforms), and JNK3 (2 isoforms) [38,47]. JNK1 and JNK2 are present in all cells, while JNK3 is expressed predominantly in the heart, brain, and testicles [46]. The JNK-dependent pathway is an important step in the pathological mechanisms of cardiac I/R injury [15,16]. Activation of JNK may vary depending on the severity and timing of oxidative stress during ischemia and/or reperfusion [44,48,49,50,51]. However, JNK activation in the heart is an early, steady, and prolonged process [18,19,20,21,52].

There are a number of reports describing the protective effect of JNK inhibitors in vivo after myocardial I/R, ex vivo in isolated hearts, and in vitro in cell lines. Indeed, administration of the JNK inhibitor SP600125 decreased the phosphorylation of JNK and reduced the lesion area and plasma concentration of lactate dehydrogenase (LDH) in rats after myocardial I/R [32,52,53]. Pretreatment with SP600125 significantly attenuated I/R-induced cardiomyocyte death, LDH leakage, and infarct size in ex vivo experiments on isolated hearts. SP600125 also improved the systolic/diastolic function of single cardiomyocytes and the whole heart with a concomitant decrease in phospho-JNK expression [54]. SP600125 also demonstrated a protective effect in I/R cellular models, including mouse cardiac myoblast H9c2 cells [55,56] and neonatal cardiac myocytes [31,57]. Similar results were obtained in vivo and ex vivo with other JNK inhibitors, including AS601245, SU3327, and SR 3306 [16,26,28,58].

An analysis of studies investigating the effects of JNK inhibitors in experimental models of MI showed that the protective effects of these compounds were assessed early after I/R. In addition, data obtained in the I/R models regarding the effects of various JNK inhibitors on cardiac contractile function were inconsistent [54,58]. The inconsistency of JNK inhibitors has been attributed to both the lack of a sufficient degree of selectivity of these compounds for JNK [14] and involvement of the JNK signaling pathway not only in MI pathogenesis but also in the chain of compensatory and protective reactions occurring in the myocardium after I/R [15,24]. Importantly, no studies on the cardioprotective effects of JNK inhibitors in the long term after myocardial I/R during the development of HF have been reported.

In previous studies, IQ-1 was profiled in a competitive binding assay for its ability to compete with an active-site-directed ligand for 91 different kinases, and the screening revealed that IQ-1 is a highly specific inhibitor of human JNKs compared with the other kinases tested [59]. Moreover, the binding affinities (K_d_) of IQ-1 toward JNK1, JNK2, and JNK3 were found to be 390, 360, and 87 nM, respectively [39], indicating that the highest inhibitory activity of IQ-1 was for JNK3, which is expressed solely in the heart and brain and may play unique roles in those tissues [60]. Furthermore, administration of 25 mg/kg IQ-1 reduced the JNK-dependent phosphorylation of c-Jun in the rat brain after focal I/R, demonstrating direct evidence of JNK3 inhibition in vivo [61].

MI in rats is the most commonly used experimental model to study HF [62,63] because the manifestations observed in rats after MI accurately reproduce the findings in humans with cardiac decompensation and substantiate the study of HF pathogenesis, pathophysiology, and treatment [64]. The period of 60 days after acute infarction in the rat I/R model represents the late phase, which is influenced by both the size of acute infarction developing after I/R and subsequent myocardial remodeling processes [20,65]. The model of MI in rats that we used allowed for reproduction of the main morphological and hemodynamic phenomena characterizing HF, which have been shown in other studies on rats with a similar timeframe of observation [66,67,68]. I/R triggers apoptosis in the myocardium, which is the main factor of LV remodeling and HF development [69,70], and JNK signaling pathways are actively involved in this process [53,71,72].

There is a favorable therapeutic window for the interruption of programmed cardiomyocyte death after MI, which spans several days after acute injury [69]. Therefore, the period of administration of IQ-1 was limited to five days. At the same time, a pronounced decrease (by more than 2.5-fold) in the infarction zone was shown in rats treated with IQ-1 at the end of this period, which reflects the manifestation of the cardioprotective activity of IQ-1 in the acute period of MI. Therefore, the period of administration, the route of administration, and the dose of IQ-1 may be considered acceptable for achieving the objectives of the study.

In the long term, a significant number of people with MI exhibit the development of LV remodeling [8,10], which usually leads to HF and often to sudden cardiac death [7,11]. HF is characterized by a decreased ejection fraction, progressive chamber dilation, proinflammatory cytokine release, apoptosis, and fibrogenesis in the MI zone and distant areas [6,7,9]. According to the morphological study, rats in the control group showed characteristic signs of HF 60 days after I/R, including a significant increase in the LV cavity, LV dilatation, and the formation of dense foci of mature connective tissue mainly in the central sections of the postinfarction zone, apparently due to the replacement of dead cardiomyocytes by fibroblasts, which is consistent with reports on myocardial remodeling after I/R [73,74]. Shifts in systemic hemodynamic parameters typical for HF were also observed in rats in the control group, including decreases in aortic pressure, stroke volume, and cardiac output. The development of pronounced systolic dysfunction was confirmed by the expected and pronounced decreases in LV systolic pressure, +dP/dt_max_, and the contractility index. Diastolic dysfunction was manifested as decreased –dP/dt_max_, increased LVP_min_, and dramatically increased LV end-diastolic pressure. These data correspond to the results obtained by other authors over a long-term period after myocardial I/R [12,66,75,76].

We report here for the first time the cardioprotective effects of a JNK inhibitor in the long term after MI. Indeed, the symptoms of HF at 60 days after MI simulation were less severe in rats treated with IQ-1. Compared with the control group, there were significant decreases in the sizes of the LV cavity and postinfarction focus, which was probably due to the better preservation of cardiomyocytes in the peripheral areas, as well as the limitation of the mature connective tissue zone. None of the animals showed the formation of an aneurysm of the anterior wall of the left ventricle. There was an improvement in the pumping function of the heart in animals treated with IQ-1, which resulted in a significant improvement in the contractile function of the myocardium compared with the control group of rats, including increased LV systolic pressure, +dP/dt max, –dP/dt max, and contractility index, as well as decreased LV end-diastolic pressure and LVP_min_. The increase in stroke volume, cardiac output, and aortic pressure were integral manifestations of HF alleviation. According to some researchers, fibrosis in the non-infarct area of the myocardium is the main cause of ventricular remodeling after MI [77,78,79]. The extracellular matrix deposition at sites remote from the infarct area can lead to cardiac stiffness, an inevitable process of myocardial remodeling that occurs in the aftermath of MI and constitutes the basis for the development of HF [79]. We analyzed the density of collagen in the zone distant from the infarct area, namely, in the interventricular septum, two months after MI and found a significant increase in the process of fibrotic scarring in control rats. This process was reduced in rats treated with IQ-1. However, the contribution of this phenomenon to the improvement in LV contractility will need to be studied in more detail.

Since IQ-1 was administered in a short course after I/R, and the administration was discontinued on day 6, the long-term attenuation of HF symptoms was clearly due to the cardioprotective effects of IQ-1 during its administration during acute MI. We believe that, in addition to the selectivity of IQ-1 as a JNK inhibitor, the cardioprotective effect of this compound may be due to its antioxidant properties. Indeed, the antioxidant properties of IQ-1 manifested themselves in limiting lipid peroxidation product accumulation in brain tissue after global cerebral ischemia [80]. Oxidative stress is an essential element of MI pathogenesis and a direct consequence of I/R [81,82] and leads to the activation of JNK [83]. JNK has been demonstrated to be involved in cell apoptosis and necrosis by increasing the production of cytotoxic reactive oxygen species [26,84,85,86].

An acute inflammatory response has been shown to occur during the first days after MI [43,87,88], and persistent inflammation can contribute to adverse post-MI LV remodeling, making inflammation an important therapeutic target for improving outcomes of MI [89]. Since JNKs play a key role in the regulation of inflammation involved in cardiomyocyte injury during I/R [15], we suggest that the anti-inflammatory activity of IQ-1 [90] contributes to the beneficial effects that we observed during MI. For example, IQ-1 suppresses the expression of the metalloproteinase (MMP)-1 and MMP-3 genes in fibroblast-like cells [59]. These proteinases play a critical role in LV remodeling [91,92]. Thus, we propose that the cardioprotective properties of IQ-1 observed in the long term (i.e., 60 days after MI) result from the anti-inflammatory effects of this compound during the acute period after I/R by limiting the scar zone and myocardial remodeling, which then manifests itself as less severe HF during the later period. However, to reveal the acute anti-inflammatory effects of IQ-1 during MI, further experimental investigation is needed.

## 5. Conclusions

In conclusion, we report the cardioprotective properties of the selective JNK inhibitor IQ-1. These properties manifested as a reduction in the infarct zone five days after acute I/R in the rat model of MI. HF was also alleviated 60 days after MI, which was confirmed by significant decreases in the scar zone, LV cavity sizes, and the volume of connective tissue in the infarct zone and the remote zone, as well as the improvement in contractile activity and the alleviation of LV diastolic dysfunction. We believe that IQ-1 may be considered a candidate molecule for the development of an effective drug with cardioprotective activity in MI. Further studies are required to elucidate the contributions of the anti-inflammatory properties of IQ-1 to the cardioprotective effect of the compound after I/R.

## Figures and Tables

**Table 1:** Effects of IQ-1 on systemic hemodynamic and cardiac contractility parameters in rats 60 days after MI.

Parameter	Group		
	Sham-Operated (n = 7)	Control (n = 11)	IQ-1 (n = 11)
Stroke volume (mL)	0.22 ± 0.02	0.15 ± 0.01 *	0.20 ± 0.01 ^#^
Heart rate (min^–1^)	381 ± 19	383 ± 10	393 ± 14
Cardiac output (mL × min^–1^)	84.8 ± 6.1	56.4 ± 3.0 *	78.7 ± 3.2 ^#^
Contractility index (s^–1^)	119 ± 1	79 ± 3 *	106 ± 2 ^#^
Aortal pressure (mm Hg)	119 ± 3	101 ± 3 *	113 ± 3 ^#^
Left ventricular systolic pressure (mm Hg)	129 ± 4	108 ± 7 *	122 ± 3 ^#^
+dP/dt_max_ (mm Hg/s)	7566 ± 124	4870 ± 363 *	6585 ± 172 ^#^
–dP/dt_max_ (mm Hg/s)	–6369 ± 152	–3295 ± 263 *	–4875 ± 160 ^#^
Left ventricular end-diastolic pressure (mm Hg)	1.4 ± 1.1	13.2 ± 1.8 *	3.9 ± 0.9 ^#^
Minimum pressure in the left ventricle (mm Hg)	–1.8 ± 1.1	10.0 ± 1.7 *	0.8 ± 1.0 ^#^

Legend: +dP/dt_max_, maximum rate of pressure rise in the LV; –dP/dt_max_, maximum rate of pressure drop in the LV. * *p* < 0.05 as compared with sham-operated animals; ^#^ *p* < 0.05 as compared with control animals.

## Data Availability

The data are contained within the article.
